# A Benefit of Being Heavier Is Being Strong: a Cross-Sectional Study in Young Adults

**DOI:** 10.1186/s40798-018-0125-4

**Published:** 2018-03-01

**Authors:** Gill A. ten Hoor, Guy Plasqui, Annemie M. W. J. Schols, Gerjo Kok

**Affiliations:** 1 Department of Human Biology, Nutrition and Translational Research in Metabolism, Maastricht University Medical Centre+, P.O. Box 616, 6200 MD Maastricht, The Netherlands; 2 Department of Work and Social Psychology, Maastricht University, P.O. Box 616, 6200 MD Maastricht, The Netherlands; 3 Department of Respiratory Medicine, Research School NUTRIM, Maastricht University Medical Centre, P.O. Box 616, 6200 MD Maastricht, The Netherlands

**Keywords:** Overweight, Body composition, Strength, Motivation, Attitude

## Abstract

**Background:**

In this study, the main hypothesis is that heavier people enjoy strength exercises more than normal-weight people, mediated by fat-free mass and muscle strength. Further, it is hypothesized that heavier people are better in strength exercises and enjoy strength exercises more compared to aerobic exercises.

**Methods:**

In a cross-sectional study, height, weight, body composition (i.e., fat mass and fat-free mass by underwater weighing), muscle strength (i.e., one-repetition maximal strength for the leg press and chest press), maximal aerobic exertion (VO_2_max) during cycle ergometry, and psychological determinants (i.e., attitudes, intentions, and self-determined motivations for strength exercises and aerobic exercises using questionnaires) were measured in 68 participants (18–30 years).

**Results:**

Significant correlations between weight/BMI and fat-free mass (index) (*r* values = .70–.80, *p* values < .001), fat-free mass and muscle strength (*r* values = .35–.55, *p* values < .05), and muscle strength and attitudes, intentions, and motivation for strength exercises were found (*r* values = .29–.43, *p* values < .05); BMI was related to psychological determinants via fat-free mass and muscle strength. Furthermore, participants with a higher BMI are significantly better in strength exercises, more intrinsically motivated, and less motivated to do strength exercises compared to aerobic exercises (all *p* values < .05). Trends in the same direction were found for the following variables: instrumental attitude, experiential attitude, and intention (*p* values < .1).

**Conclusions:**

Strength exercises could be more appropriate for heavier people and might therefore be a valuable component in physical activity programs for people who are overweight or obese.

**Electronic supplementary material:**

The online version of this article (10.1186/s40798-018-0125-4) contains supplementary material, which is available to authorized users.

## Key Points

- Heavier people not only have more fat mass but also more fat-free mass, likely making them stronger (in absolute sense) compared to normal-weight people.
- Heavier people are more positive about strength exercises compared to (1) normal-weight people and (2) aerobic exercises.
- Performing strength exercises has beneficial effects on body composition and, with that, on metabolic and cardiovascular health.

## Background

Obesity is a worldwide problem with high costs to society and well-being [1, 2]. Being physically active can prevent and decrease obesity [3] but is often challenging for people who are overweight or obese [4–6]. In this study, we try to bridge the gap between biological and psychological insights in the management of obesity, by examining the putative physiological and psychological benefits of strength exercises for heavier people [4–6]. People who are overweight do not only have more fat mass but also more fat-free mass [7]. With that, people who are overweight or obese are likely to have more muscle mass and to be stronger compared to people who are not overweight. Compared to aerobic exercises, strength exercises are easier for people who are overweight, and therefore, compliance to an exercise program focused on strength exercises is greater [8]. By being better in strength exercises than aerobic exercises, people who are overweight might be more positive about strength exercises compared to normal-weight people, and with that, long-term behavior change may be achieved [4–6]. Additionally, performing strength exercises has beneficial effects on overweight or obese people’s body composition and, with that, on their metabolic and cardiovascular health [1]. In this study, we cross-sectionally test the “chain of assumptions” that (1) heavier people have more fat mass and more fat-free mass, (2) they are stronger and better in strength exercises, and (3) they have more positive associations with strength exercises. This chain resulted in two hypotheses. The main hypothesis is that (1) heavier people are more positive about strength exercises compared to normal-weight people, because they have more fat-free mass and a higher muscle strength. Further, it is hypothesized that (2) people who are heavier are not only better in strength exercises but also more positive about strength exercises compared to aerobic exercises.

## Methods

Following pleas for full disclosure [9, 10], all research materials and data are combined in Additional files 1, 2, 3 and 4. This study was approved by the Ethics Committee of the Maastricht University Medical Center+ (NL43929.068.13/METC 13-3-018) and conforms with the Code of Ethics of the World Medical Association (Declaration of Helsinki).

### Participants

A total of 70 participants (18–30 years of age) were recruited among students of Maastricht University. Two participants did not return on day 2 of the study and were therefore excluded from the analyses. To get a better range in body mass index (BMI) and body composition, we advertised the study using flyers, including a statement that we were especially interested in students with a BMI > 24. All participants were screened for good health using a general medical questionnaire (see Additional files 1 and 3) to ensure that participants were able to perform exercises. Participants were excluded when they had any condition that prevented them from performing the exercise protocols (e.g., sports injuries or severe asthma). Prior to participation, written informed consent was obtained.

### Procedure and Measures

Participants were invited to participate in a 2-day cross-sectional study. Participants that expressed their interest (by responding to the advertisement) and were found eligible (based on the medical screening questionnaire; see Additional files 1 and 3) were invited to visit the university on 2 days, with an 8–10-day interval.

For day 1 (that always started between 8 and 8.30 am), the participant was asked to refrain from any high intensity physical exercise 24 h prior to the testing and to come to the laboratory in a fasted state (overnight fast from 10 pm onwards). In the Metabolic Research Unit at Maastricht University, Maastricht, The Netherlands, height and weight of the participant were measured, and body composition was assessed using underwater weighing (see section “Body Composition”). Subsequently, participants were asked to eat a light breakfast (such as one slice of bread with cheese). One hour after having eaten a light breakfast, cardiovascular fitness was assessed with a maximal exercise test (VO_2_max test; see section “Maximal Aerobic Exertion Test (VO_2_max)”) on a bicycle ergometer. Approximately 1 h after the VO_2_max testing, the participant performed a familiarization session with the exercise equipment to estimate one-repetition maximum (1RM; see also section “Maximal Strength Exertion Test (1RM Test)”) strength in the fitness lab at Maastricht University. During the familiarization session, proper lifting techniques were demonstrated for leg press and chest press exercises by a trained instructor.

At day 2, 8–10 days after day 1, the actual 1RM testing took place in the fitness lab at Maastricht University (see section “Maximal Strength Exertion Test (1RM Test)”). Subsequently, the participant was asked to perform a series of standardized strength and aerobic exercise activities (see “Standardized Exercise Protocols”). Directly after each exercise activity (both the strength and aerobic exercise), a questionnaire was filled out to measure social cognitive determinants (see “Questionnaires”). All data were collected by the first author and three research assistants (always under close supervision of the first author; see “Acknowledgements”). For the biological measurements, validated and reliable protocols were used (see sections “Body Composition” and “Maximal Strength Exertion Test (1RM Test)”). The exercise protocol (section “Standardized Exercise Protocols”) was always performed under supervision of two researchers to ensure proper execution. The psychological questionnaires were based on validated constructs (see section “Questionnaires”).

**Fig. 1:**
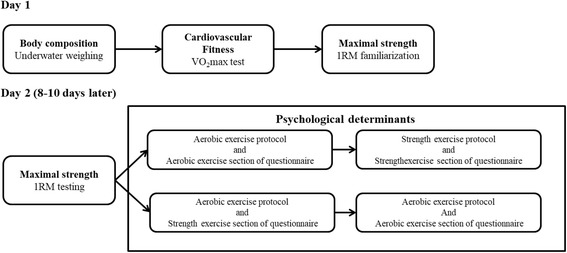
Flowchart of the study protocol

After completion of both days, participants received a gift voucher and travel expenses. Participants from the Faculty of Psychology and Neuroscience at Maastricht University were able to choose between the gift vouchers, or participation credits (part of the psychology bachelor curriculum) (see also Fig. 1 and Additional file 4  for a clear overview of the study protocol).

#### Body Composition

Anthropometric measurements, body mass, and height were taken in the morning after an overnight fast on day 1 of testing. Body mass was measured on an electronic scale to the nearest 0.01 kg. Height was measured to the nearest 0.1 cm. Body composition was calculated based on underwater weighing with simultaneous measurement of residual lung volume using the helium dilution technique (Volugraph 2000, Mijnhardt). For this measurement, participants are in a fasted state and are completely submerged under water, for approximately 90 s to measure their weight under water, while breathing oxygen through a mouthpiece. The measurement was repeated three times. Body volume was calculated using the following formula: ((body mass_dry_ − body mass_under water_) / water density) − lung volume. Body density was derived from body weight and body volume, which was used to calculate fat mass and fat-free mass by the Siri equation [11].

#### Maximal Aerobic Exertion Test (VO_2_max)

Cardiovascular fitness was assessed with an incremental test on a bicycle ergometer using the protocol of Kuipers et al. [12]. During this test on day 1, oxygen consumption (VO_2_) and CO_2_ production were measured continuously (Omnical, Maastricht University) and heart rate was monitored using a polar heart rate monitor (RS400, Polar Electro, Kempele, Finland; watch worn by the instructor). After a warming up of 5 min at 100 watts (W) for men and 75 W for women, the workload increases with 50 W every 2.5 min. When one’s heart rate reached a value of 35 beats per minute (bpm) below the age predicted maximal HR (220 bpm − age) or the respiratory quotient (RQ = CO_2_ production / oxygen consumption) exceeded 1, workload was increased with 25 W (instead of 50 W) every 2.5 min until exhaustion. VO_2_max was presented relative to fat-free mass (ml/kg fat-free mass/min).

#### Maximal Strength Exertion Test (1RM Test)

Approximately 1 h after VO_2_max testing on day 1, participants performed a familiarization session with the exercise equipment to estimate 1RM strength. During the familiarization session, proper lifting technique was demonstrated for leg press and chest press exercises. Guided-motion exercise machines (one for leg press, one for chest press) were used to establish safe and proper lifting. Maximum strength was estimated in all participants using the multiple-repetition testing procedure [13]. In a separate session (8–10 days later; day 2), the actual 1RM testing took place. After warming up (5 min on light load on cycle ergometer), two sets of 12 repetitions were performed on the exercise machines at a light load (15 and 25 kg on the chest press and 70 and 80 kg on the leg press, for female and male participants, respectively). Next, the load was set at 95% of the estimated 1RM and one repetition was performed. Thereafter, the load was increased by 2.5–5.0% after each successful lift until the participant was able to perform a maximum of one repetition [14].

#### Standardized Exercise Protocols

On day 2, standardized exercise protocols were carried out before each questionnaire (i.e., a strength exercise protocol before the strength exercise questions, and an aerobic exercise protocol before the questions about aerobic activities). The goal of the protocols was to let participants experience strength and aerobic exercises to improve the validity of the questionnaires that were filled out immediately afterwards (to measure the so-called “experiential” attitudes and motivations). In both the aerobic and strength exercise protocols, two different exercises were included to ensure that people did not answer questions about “cycling” but about “aerobic exercises.” The duration of the strength and aerobic fitness protocol was similar (~ 20 min), and the order of the protocols was randomized to control for a possible order effect. For both protocols, we chose to work on 70% of their maximum. A higher percentage unnecessarily increased the chances of injury/anaerobic training, while a lower percentage would be too low (i.e., warming up or too low exertion). The strength protocol was based on the two different 1RM tests (for leg press and chest press). The 70% of maximal strength on the leg press exercise and chest press exercise was calculated. After a 5-min warmup (75 W; bicycle ergo meter—to minimize the chance for injuries), participants were asked to do three sets of 8–10 repetitions on the chest press apparatus and three sets of 8–10 repetitions on the leg press apparatus. Between each set, there was a 2-min break. Between the leg press and the chest press, there was a 5-min transition time break. Also, the order of the chest press and leg press was randomized to control for a possible order effect.

The aerobic exercise protocol (conducted on day 2) was based on maximal heart rate and maximal workload measured during the VO_2_max test conducted on day 1, and included both cycling and running. After a 5-min warmup (75 W, bicycle ergo meter), participants were asked to cycle for 10 min on 70% of their Wmax (70 RPM; the same as during the VO_2_max test) and to run 3 × 3 min at 70% of their maximal heart rate on a treadmill (no inclinations, and the speed was continuously adjusted by the instructor to keep the participant at 70% of their maximal heart rate). The running was introduced to also have two different aerobic exercises. Between the three sets of running, participants walked for 1 min. Between the cycling and running, there was a 5-min transition time break. The order of the cycling and running was counterbalanced as well.

#### Questionnaires

Participants completed a questionnaire, based on the reasoned action approach [15] and the self-determination theory [16]. This questionnaire included specific and general questions about resistance and aerobic exercises and was divided accordingly into two sections for completion (i.e., resistance exercise questions were answered following completion of the strength exercise protocol, and the aerobic exercise questions were answered following completion of the aerobic exercise protocol; see also Fig. 1). Filling out the questionnaires took about 3–5 min per stage. The measured constructs are as follows: (1) instrumental attitudes (cognitive feelings about exercises), (2) experiential attitudes (affective feelings about the exercises), (3) intentions (whether the participant intends to do the specific exercise in the near future), (4) intrinsic motivation (how fun the exercise is), and (5) a-motivation (no motivation to do the specific exercise at all). All items were rated on a 7-point Likert scale. Scores on items that measured the same construct were averaged into one scale where internal consistency was sufficient (*α* > .60). One item was deleted (“After doing this exercise, I’m satisfied no matter what my performance is”) for both aerobic and strength exercises, as reliability analysis showed low-scale reliability when this item was added to the intrinsic motivation construct. Scores were recoded such that a higher score reflected a higher value on the variable (see also Table 1 for all exact items, scoring, and Cronbach’s alpha).

**Table 1:** Attitudes, intentions, and motivations related to aerobic and resistance exercises (*n* = 68)

Determinant	Questions	Rating (1–7)	Cronbach’s *α*	
			Aerobic	Strength
Instrumental attitude	How good do you think this exercise is?	Very bad–very good	.67	.84
	How healthy do you think this exercise is?	Very unhealthy–very healthy		
	To me, strength exercises are	Very unimportant–very important		
	How useful do you think strength exercises are?	Not useful at al–very useful		
	How healthy are strength exercises for you?	Very unhealthy–very healthy		
Experiential attitude	How did the exercise feel?	Very unpleasant–very pleasant	.80	.89
	What did you think of the exercise?	Very boring–very exciting		
	I think strength exercises in general are	Very unpleasant–very pleasant		
	I think strength exercises in general are	Very boring–very exciting		
Intention	I will do strength exercises in the future	Totally disagree–totally agree	.96	.97
	I am planning to do strength exercises in the future	Totally disagree–totally agree		
	I expect to do strength exercises in the future	Totally disagree–totally agree		
Intrinsic motivation	The exercise I just did is something I would like to do in my free time	Totally disagree–totally agree	.83	.93
	I would like to do strength exercises in my free time	Totally disagree–totally agree		
	I enjoy doing strength exercises	Totally disagree–totally agree		
A-motivation	I am not made for this exercise	Totally disagree–totally agree	.85	.83
	This exercise did not feel right for me	Totally disagree–totally agree		
	I will never be good at strength exercises	Totally disagree–totally agree		
	I am not suitable for strength exercises	Totally disagree–totally agree		

The shown answers for the questions in this table are for the strength questions. The same questions were asked for aerobic exercises (i.e., the word “strength” was replaced by the word “aerobic”)

### Data Analysis

IBM SPSS statistics and Excel were used to analyze the data (see also Additional file 2). Frequencies (*n*), means (*M*), and standard deviations (SD) were calculated to provide an overall picture of the sample. Paired sample *t*-tests were conducted to calculate differences between male and female participants. Pearson’s correlations were calculated to examine associations between the various determinants. We tested the direct and indirect associations linking BMI scores with psychological constructs regarding strength exercises using the PROCESS software including the bootstrapping method with bias-corrected confidence estimates (see also Fig. 3) [17, 18]. Bootstrapping, a non-parametric sampling procedure, was used to assess the significance of indirect effects. In the present study, the 95% confidence interval of the indirect effects was obtained with 5000 bootstrap resamples; results are statistically significant when 95% confidence intervals did not include zero. To compare correlations of BMI with strength and BMI with aerobic outcomes, first, the difference in Fisher’s *z* was calculated. Based on the *z* score of this difference, *p* values were estimated [19].

## Results

A total of 68 participants participated in this study (BMI ranged from 18 to 38). Male (*n* = 33) and female (*n* = 35) participants did not differ in age, BMI, VO_2_max, or self-reported physical activity (all *p* values > .05), but male participants were taller, heavier, and stronger. Female participants had a higher fat mass compared to male participants (see Table 2). Self-reported activity levels ranged from very high (14 h/week) to not active at all (mean [SD], 4 h/week [3 h]; median 3.5 h; not reported in the table).

**Table 2:** Study sample characteristics

	Total	Male	Female	*t* (*df*)	*p*	95% CI
	*M* (SD)					
	*N* = 68	*N* = 33	*N* = 35			
Age (years)	23 (3)	23 (3)	23 (3)	0.6 (66)	.51	− 1.03 to 2.04
Height (cm)	175.0 (8.4)	181.1 (6.1)	169.3 (6.0)	− 7.9 (66)	<.001	− 14.66 to − 8.75
Weight (kg)	72.0 (12.6)	79.2 (10.7)	65.2 (10.4)	− 5.5 (66)	<.001	− 19.13 to − 8.95
BMI (kg/m^2^)	23.4 (3.2)	24.2 (3.3)	22.6 (3.0)	− 2.0 (66)	.05	− 3.05 to − 0.01
Fat mass (kg)	17.3 (7.3)	15.0 (7.3)	19.5 (6.7)	2.6 (66)	.01	1.09–7.88
Fat-free mass (kg)	54.6 (11.2)	64.2 (6.8)	45.7 (5.8)	− 12.1 (66)	<.001	− 21.57 to 15.47
VO_2_max (ml/min/fat-free mass)	54.3 (6.8)	55.0 (6.4)	53.6 (7.2)	− 0.8 (66)	.40	− 4.80 to 1.94
Self-reported physical activity	4.1 (2.9)	4.2 (2.7)	3.9 (3.2)	0.48 (66)	.64	− 1.09 to 1.77
Leg press (1RM)	196.8 (50.6)	234.2 (35.5)	161.4 (34.8)	− 8.5 (66)	<.001	− 89.85 to − 55.78
Chest press (1RM)	70.7 (29.9)	95.8 (21.4)	46.9 (11.9)	− 11.6 (49)	<.001	− 57.39 to − 40.42
Aerobic exercises						
Instrumental attitude	6.3 (0.5)	6.2 (0.6)	6.3 (0.4)	1.3 (66)	.21	− 0.09 to 0.41
Experiential attitude	4.9 (1.0)	5.0 (1.0)	4.9 (1.1)	− 0.1 (66)	.92	− 0.53 to 0.48
Intention	5.7 (1.3)	5.6 (1.4)	5.8 (1.3)	0.6 (66)	.57	− 0.45 to 0.82
Intrinsic motivation	5.4 (1.2)	5.4 (1.1)	5.4 (1.3)	0.1 (66)	.96	− 0.56 to 0.60
A-motivation	2.2 (1.0)	2.0 (1.1)	2.3 (1.0)	0.9 (66)	.36	− 0.28 to 0.74
Strength exercises						
Instrumental attitude	5.3 (0.9)	5.4 (0.9)	5.1 (0.9)	− 1.3 (66)	.21	− 0.73 to 0.16
Experiential attitude	4.3 (1.3)	4.5 (1.3)	4.1 (1.3)	− 1.3 (66)	.20	− 1.04 to 0.22
Intention	4.6 (1.9)	5.1 (1.9)	4.2 (1.8)	− 1.9 (66)	.06	− 1.75 to − 0.04
Intrinsic motivation	4.4 (1.8)	4.7 (1.8)	4.1 (1.7)	− 1.5 (66)	.14	− 1.51 to 0.21
A-motivation	2.8 (1.3)	2.5 (1.3)	3.1 (1.2)	2.2 (66)	.04	0.05 to 1.26

### Being Heavier Means More Fat-Free Mass, Means Stronger, and Means More Positive Results on Psychological Constructs

**Fig. 2:**
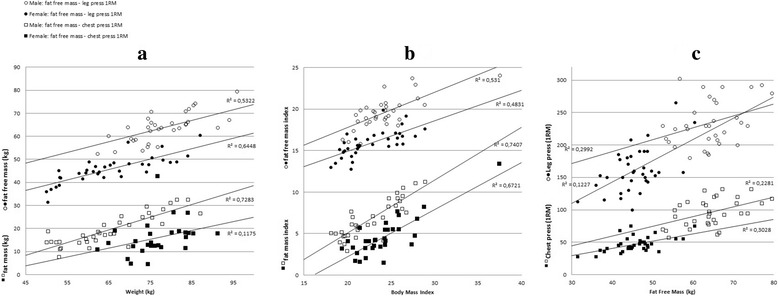
Correlational analyses. **a** Correlations between weight and fat mass and between weight and fat-free mass for male and female participants, separately. **b** Correlations between BMI and fat mass index and between BMI and fat-free mass index for male and female participants, separately. **c** Correlations between fat-free mass and strength measures for male and female participants, separately

Correlational analyses revealed significant correlations between weight and fat mass (*r* = .85 for female and *r* = .78 for male participants, all *p* < .001), and BMI (weight adjusted for height), and fat mass index (fat mass adjusted for height; *r* = .86 for female and *r* = .82 for male participants, all *p* < .001; see Fig. 2a). Weight and BMI were also highly correlated with the fat-free mass and fat-free mass indices, respectively (*r* values ranging from .70 to .80, all *p* < .001; see Fig. 2b). Participants with a higher fat-free mass had a significantly higher chest press 1RM (*r* = .55 for female and *r* = .48 for male participants, all *p* < .005) and leg press 1RM (*r* = .55 for female, *p* = .001, and *r* = .35 for male participants, *p* = .046; see Fig. 2c). Finally, a combined strength score (sum of leg press 1RM and chest press 1RM) was positively correlated with instrumental attitude (*r* = .29, *p* = .02), experiential attitude (*r* = .31, *p* = .008), one’s intention to start with strength exercises (*r* = .35, *p* = .02), and intrinsic motivation (*r* = .33, *p* = .007). An expected negative correlation was found with a-motivation (*r* = − .43, *p* < .001; see Table 3).

**Table 3:** Correlations between strength and psychological outcomes (*n* = 68)

	Strength*		Strength*	
	*r*	*p*	*r*	*r*
			Female	Male
Instrumental attitude (1–7)	.29	.02	.22	.32
Experiential attitude (1–7)	.32	.008	.31	.34
Intention (1–7)	.35	.003	.19	.37
Intrinsic motivation (1–7)	.33	.007	.23	.37
A-motivation (1–7)	− .43	<.001	− .29	− .46

*The strength measure is the sum score of chest press 1RM and leg press 1RM

**Fig. 3:**
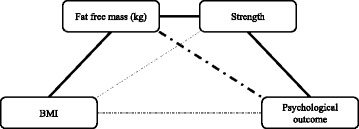
Model for testing the indirect relations of BMI with psychological outcomes. There was neither an direct effect of BMI on psychological outcomes nor an indirect effect of BMI on psychological outcomes via strength. Limited indirect effects of BMI on psychological outcomes were found via fat-free mass. Significant effects of BMI on psychological outcomes were found when fat-free mass and strength were added to the model

There was no direct effect of BMI on attitudes, intention, or motivations (*p* values range from .44 to .95; see Fig. 3 and Table 4). Indirect effects of BMI on all psychological outcomes were found via fat-free mass and the combined strength score. No indirect effect from BMI to psychological outcomes was found via strength only. BMI had an indirect effect on experiential attitude (*β* = − .08, SE = .05, CI SE = − .18 to − .01) and a-motivation (*β* = .09, SE = .04, CI SE = .01–.19) via fat-free mass (see Fig. 3 and Table 4).

**Table 4:** Outcomes of the mediation analyses (*n* = 68)

	*β*	SE	CI SE
Direct effect of BMI on psychological outcome			
Instrumental attitude	.03	.04	− .05 to .11
Experiential attitude	− .01	.05	− .12 to .10
Intention	− .00	.08	− .16 to .15
Intrinsic motivation	− .01	.08	− .14 to .16
A-motivation	− .02	.05	− .11 to .09
Indirect: BMI − fat-free mass − psychological outcome			
Instrumental attitude	.03	.02	− .11 to .01
Experiential attitude*	*− .08*	*.05*	*− .18* to *− .01*
Intention	− .04	.06	− .18 to .06
Intrinsic motivation	− .09	.07	− .25 to .01
A-motivation*	*.09*	*.04*	*.01–.19*
Indirect: BMI − strength − psychological outcome			
Instrumental attitude	.01	.01	− .00 to .05
Experiential attitude	.02	.02	− .01 to .08
Intention	.03	.03	− .01 to .10
Intrinsic motivation	.03	.03	− .01 to .10
A-motivation	− .03	.03	− .09 to .01
Indirect: BMI − fat-free mass − strength − psychological outcome			
Instrumental attitude*	*.05*	*.03*	*.02–.12*
Experiential attitude*	*.10*	*.04*	*.04–.21*
Intention*	*.11*	*.05*	*.2–.25*
Intrinsic motivation*	*.13*	*.06*	*.05–.28*
A-motivation*	*− .12*	*.04*	*− .23* to *− .06*

*significant pathways

### Strength Versus Aerobic Exercises

To examine whether heavier people are relatively better in strength exercises than aerobic exercises compared to normal-weight people, correlations between BMI and strength outcomes and BMI and aerobic outcomes were calculated. Based on these correlations, a difference in Fisher’s *z* was calculated and *p* values were estimated [19]. Comparing aerobic and strength variables shows that when participants have a higher BMI, they are significantly better in strength exercises compared to aerobic exercises (Fisher’s *z* = .91, *p* < .001), more intrinsically motivated (Fisher’s *z* = .46, *p* < .008), and less a-motivated (Fisher’s *z* = .40, *p* < .02) for strength exercises compared to aerobic exercises. For the variables instrumental attitude, experiential attitude, and intention, the directions of the relations were the same, but these variables were not significant (*p* values ranged from .06 to .08) (see Table 5).

**Table 5:** Comparison of correlations between BMI and aerobic variables and between BMI and strength variables

	BMI	Fisher’s *z* difference	*p*
Max strength	.49	.91	<.001
VO_2_max	− .36		
Instrumental attitude			
Strength	.20	.33	.06
Aerobic	− .13		
Experiential attitude			
Strength	.09	.30	.08
Aerobic	− .21		
Intention			
Strength	.15	.32	.06
Aerobic	− .17		
Intrinsic motivation			
Strength	.14	.46	.008
Aerobic	− .31		
A-motivation			
Strength	− .20	.40	.02
Aerobic	.19		

## Discussion

We (1) confirmed that heavier people have a higher fat-free mass compared to normal-weight people. This is in line with biological insights [7]. Additionally, (2) we have shown that people with a higher fat-free mass are stronger (in absolute sense) and are better in strength exercises than in aerobic exercises. We have also confirmed that (3) mastery experiences (in this case, resulting from successfully engaging in strength exercises as opposed to aerobic exercises) are related to more positive psychological outcomes. This observation is in line with psychological insights [20–23]. As hypothesized, we (4) have shown that heavier people are more positive about strength exercises compared to normal-weight people, via fat-free mass and muscle strength. Moreover, (5) heavier people are better in strength exercises and are more positive about strength exercises compared to aerobic exercises.

To the best of our knowledge, this is the first time that this chain of relationships has been demonstrated empirically, thereby bridging the gap between biological and psychological insights. In light of these results, new exercise interventions for people with overweight or obesity could be developed, concentrating on biological strengths and using psychological principles and techniques to make them more aware of their strengths [6]. Additionally, for long-term behavior and health changes, new interventions might benefit from focusing (and giving feedback; [24]) on body composition instead of weight.

There are some limitations that should nuance the drawn conclusions. Most of the study participants are university students who volunteered to participate which might limit the generalizability of our study results. The self-reported physical activity level was higher than 45-year-old parents (2.8 h/week) but lower than 13-year-old children (5.3 h/week) [25]. The sample size is relatively small, but the used measures were accurate. The BMI range was limited, making more research necessary among a broader BMI range. Cross-sectional data instead of longitudinal data was gathered. With that, we were not able to show causality. Two additional questions might be (1) whether the exercise protocols adequately encompass what strength and aerobic exercises are and (2) whether the (possibly different) training loads of the two different exercises might have influenced the results.

To ensure that we actually worked with strength and aerobic exercises, we used exercises that are generally used in our gold standard maximal strength tests and aerobic tests (the additional running is also used very often in VO_2_max tests; see, e.g., [26]. For the protocols, we limited this to 70% of the maximum and ensured that the duration (including rest periods) was similar for both exercises. In future research, it might be helpful to add an effort perception scale to measure the perceived intensity of the protocols. For our (correlational) research question, it is unlikely that difference in training protocols influences the direction of our outcomes or conclusions (they could only have weakened the effects at most in the hypothetical case that there would have been an “ideal” training intensity). However, most of our results were significant and in the right direction.

The definition of being “heavier” is based on either a high weight or BMI, suggesting that someone is less healthy compared to someone with a normal weight or BMI. However, an increased weight or BMI is not a very reliable tool to evaluate body composition and, with that, individual (metabolic) health [27]. Therefore, to examine the statement “heavier means more fat-free mass,” we reported not only correlations of fat-free mass with weight, and fat-free mass index with BMI, but also correlations of fat mass with weight and fat mass index with BMI.

## Conclusions

In conclusion, a benefit of being overweight is being strong. Strength exercise interventions might have the ability to make people who are overweight more motivated to be physically active on the long term. They might improve long-term health by improving one’s body composition (and energy balance, insulin sensitivity, blood pressure, cholesterol level, motor skills, and the chances on cardiovascular disease) [28–31]. In short, strength exercises might contribute to the management of obesity. With interventions focusing on strength exercises, the obesity problem per se will not be solved, but such programs might positively contribute to obesity-related health issues.

## Additional files

Additional file 1: READ ME – medical screening questionnaire for the study "A benefit of being heavier is being strong: a cross-sectional study in young adults". (ZIP 32 kb)

Additional file 2: Data collected for the study "A benefit of being heavier is being strong: a cross-sectional study in young adults". (XLSX 62 kb)

Additional file 3: Dutch medical screening questionnaire for the study "A benefit of being heavier is being strong: a cross-sectional study in young adults". (XLSX 67 kb)

Additional file 4: Measurement protocol for the study "A benefit of being heavier is being strong: a cross-sectional study in young adults". (DOCX 233 kb)

## Abbreviations

1RM: One-repetition maximum (strength test)
BMI: Body mass index
bpm: Beats per minute
HR: Heart rate
VO_2_max: Maximal aerobic exertion (exertion test)
