# Supplementary material for: A Benefit of Being Heavier Is Being Strong: a Cross-Sectional Study in Young Adults
Source: Sports Med Open. 2018 Mar 1;4:12. doi: 10.1186/s40798-018-0125-4 (PMC5833324; doi:10.1186/s40798-018-0125-4)
Supplement: Supplementary file 1 — READ ME – medical screening questionnaire for the study "A benefit of being heavier is being strong: a cross-sectional study in young adults". (ZIP 32 kb) [file 40798_2018_125_MOESM1_ESM.zip › Additional file 1. READ ME - med screening Q.docx]

Additional file 1. READ ME – medical screening questionnaire.

The medical screening questionnaire was a short questionnaire, including personal characteristics (Height, weight, gender, age), lifestyle (smoking behavior, medicine use, exercise behavior), personal health ([history of] medical conditions and treatment), and familiar health. When a participant was not in good health, we would have discussed this with a medical doctor related to the project. Based on the answers on this questionnaire, there was no need to discuss this with a medical doctor.
