# Supplementary material for: A Benefit of Being Heavier Is Being Strong: a Cross-Sectional Study in Young Adults
Source: Sports Med Open. 2018 Mar 1;4:12. doi: 10.1186/s40798-018-0125-4 (PMC5833324; doi:10.1186/s40798-018-0125-4)
Supplement: Supplementary file 4 — Measurement protocol for the study "A benefit of being heavier is being strong: a cross-sectional study in young adults". (DOCX 233 kb) [file 40798_2018_125_MOESM4_ESM.docx]

PPNR:

***“How good are you?***

***Find out about your physical fitness”***

The participant ___ is informed orally.

___ has signed the informed consent.

___ has filled in the medical screening questionnaire.

**UNDERWATER WEIGHING**

Date: …………….

Time: ……………

| Length (m) |  |  |  |  |  |  |
| --- | --- | --- | --- | --- | --- | --- |
| Dry weight (kg) |  |  |  |  |  |  |
| BMI |  |  |  |  |  |  |
| Weight of bed (before) (kg) |  |  |  |  |  |  |
| Weight of bed (after) (kg) |  |  |  |  |  |  |
|  |  |  |  |  |  |  |
|  | Measure | | | | | |
|  | 1a | 1b | 2a | 2b | 3a | 3b |
| Time |  |  |  |  |  |  |
| Water temperature (ºC) |  |  |  |  |  |  |
| FRC |  |  |  |  |  |  |
| Extra in (-) /out (+) |  |  |  |  |  |  |
|  |  |  |  |  |  |  |
| **Percentage Fat Mass:** |  |  |  |  |  |  |
| **Percentage Fat Free Mass:** |  |  |  |  |  |  |
| **Kg Fat Mass:** |  |  |  |  |  |  |
| **Kg Fat Free Mass:** |  |  |  |  |  |  |

Physical activity exercises are activities that requires physical exertion, especially when performed to develop or maintain fitness.

1. How many times do you exercise per week? ______ times.
2. How much time do you spend exercising per week? ______ hours.
3. Nationality:

**
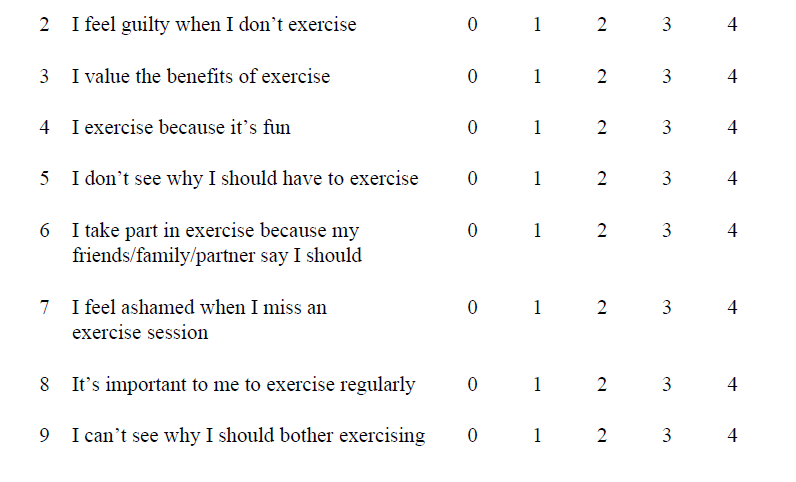

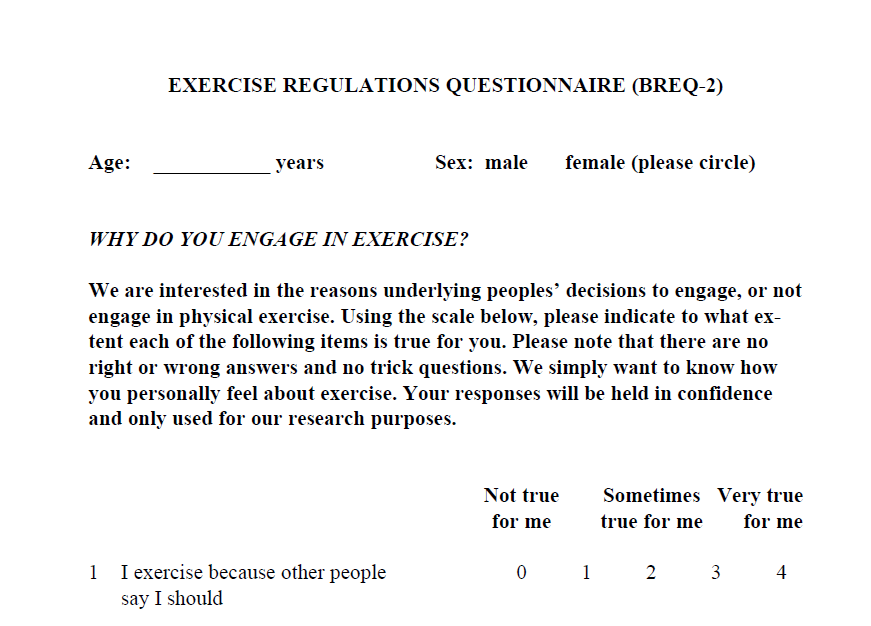
**

**
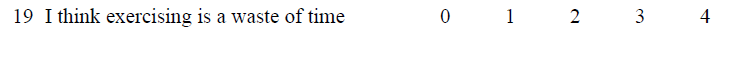

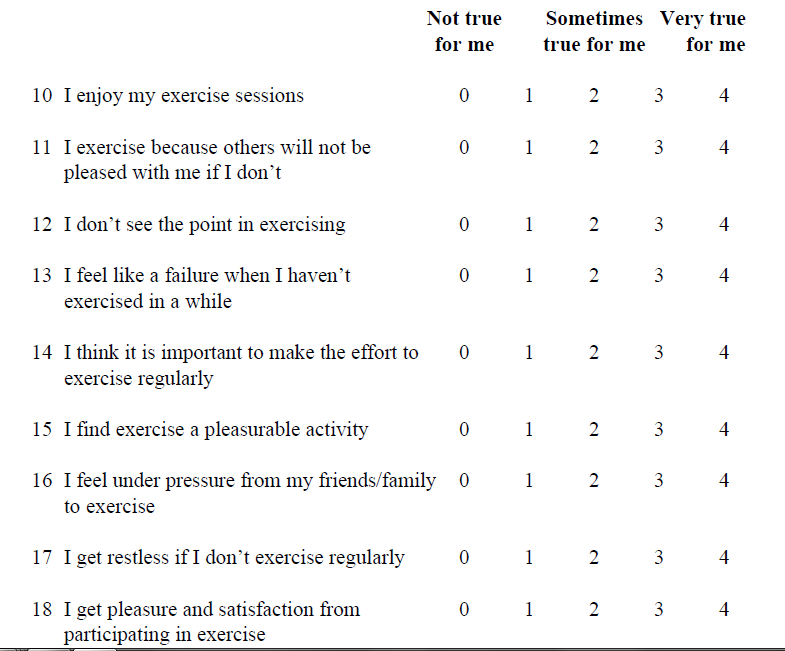
**

**Thank you. Please wait for instructions**

**VO2Max**

Date: …………….

Time: ……………

| **Time** | **W** | **VO2** | **VCO2** | **HR** |
| --- | --- | --- | --- | --- |
| **0.00** |  |  |  |  |
| **5.00** |  |  |  |  |
| **7.30** |  |  |  |  |
| **10.00** |  |  |  |  |
| **12.30** |  |  |  |  |
| **15.00** |  |  |  |  |
| **17.30** |  |  |  |  |
| **20.00** |  |  |  |  |
| **22.50** |  |  |  |  |
| **25.00** |  |  |  |  |

End time: …………………

VO2max: …………………

Wmax: ……………………

HRmax: ……………………

**Estimating IRM: Test 1**

Date: …………….

Time: ...………….

### Leg press settings:

Knee angle: 70 °, feet positioned at the upper footboard

Start position: ...... cm.

Shoulder rests: ......

|  | **Weight** | **Reps** |
| --- | --- | --- |
| Warm Up 1 (10-15 reps) |  |  |
| **Max Test 1** (4-8 reps) |  |  |
| **Max Test 2** (4-8 reps) |  |  |
| **Max Test 3** (4-8 reps) |  |  |
|  |  |  |
|  |  |  |
|  |  |  |

### Chest press:

Seat height: ...... cm

|  | **Weight** | **Reps** |
| --- | --- | --- |
| Warm Up 1 (10-15 reps) |  |  |
| **Max Test 1** (4-8 reps) |  |  |
| **Max Test 2** (4-8 reps) |  |  |
| **Max Test 3** (4-8 reps) |  |  |
|  |  |  |
|  |  |  |
|  |  |  |

___ **Accelerometer** is provided.

**1RM: Test 2**

Date: ...........................

Time: ...........................

### Leg press settings:

Knee angle: 70 °, feet positioned at the upper footboard

Start position: ...... cm.

**Calculated 1RM:**

Shoulder rests: ......

|  | **Calculated Weight** | **Applied Weight** | **Reps** |
| --- | --- | --- | --- |
| Warm Up 1 (50%, 5-10 reps) |  |  |  |
| Warm Up 2 (70%, 3-5 reps) |  |  |  |
| **Max Test 1** (95%, 1 rep) |  |  |  |
| **Max Test 2** (+5%, 1 rep) |  |  |  |
| **Max Test 3** (+5%, 1 rep) |  |  |  |
| **Max Test 4** (+5%, 1 rep) |  |  |  |
| **Max Test 5** (+5%, 1 rep) |  |  |  |
|  |  |  |  |

**Chest press:**

**Calculated 1RM:**

Seat height: position ......

|  | **Calculated Weight** | **Applied Weight** | **Reps** |
| --- | --- | --- | --- |
| Warm Up 1 (50%, 5-10 reps) |  |  |  |
| Warm Up 2 (70%, 3-5 reps) |  |  |  |
| **Max Test 1** (95%, 1 rep) |  |  |  |
| **Max Test 2** (+5%, 1 rep) |  |  |  |
| **Max Test 3** (+5%, 1 rep) |  |  |  |
| **Max Test 4** (+5%, 1 rep) |  |  |  |
| **Max Test 5** (+5%, 1 rep) |  |  |  |
|  |  |  |  |

**Chest press 70%:**

**Leg press 70%:**

**Protocol aerobic exercises**

Date: ..........................

**70% W_max_:**

Time: ..........................

1. **Cycling** (3x3 min @ 70 % of W_max,_ 1 min break @ 75W )
   1. Was the participant capable of completing the exercise according to the guidelines?
   2. Did the researcher have to adjust the guidelines?

**70% HR_max_:**

1. **Running** (3x3 minutes @ 70% of HR_max_ ( range 15 beats), 1 minute break @ pace 4)
   1. Was the participant capable of completing the exercise according to the guidelines?
   2. Did the researcher have to adjust the guidelines?

**Protocol resistance exercises**

Date: ..........................

Time: ..........................

1. **Leg press**: 3x10 reps at **70% of determined 1RM = __________**
   1. Set 1: ........ repetitions
   2. Set 2: ........ repetitions
   3. Set 3: ........ repetitions

Remarks:

1. **Chest press**: 3x10 reps at **70% of determined 1RM = ___________**
   1. Set 1: ........ repetitions
   2. Set 2: ........ repetitions
   3. Set 3: ........ repetitions

Remarks:

**If the participant is not able to complete the sets try to help him out with the exercise!**

**Questionnaire – Performing in aerobic & strength exercises**

**Instructions**

Read the questions carefully and answer all questions. When the preferred answer is not one of the answering options, choose the best fitting answer

The questions have a scale from 1 to 7, where you have to cross the box for the answer that is most appropriate for your **current situation**.

For example: if you’re asked to judge the weather in Maastricht, you should interpret the scale as following:

The weather in Maastricht is

**very very**

**good** 0 0 0 0 0 0 0 **bad**

very somewhat not good, somewhat very

good good good not bad bad bad bad

(if you think the weather is very good)

The weather in Maastricht is

**very very**

**good** 0 0 0 0 0 0 0 **bad**

(if you think the weather is bad)

The weather in Maastricht is

**very very**

**good** 0 0 0 0 0 0 0 **bad**

(if you think the weather is neutral, or if you don’t have a specific opinion)

The weather in Maastricht is

**very very**

**good** 0 0 0 0 0 0 0 **bad**

**By filling out the questionnaire, please make sure that you:**

*** answer all questions**

*** don’t give more than one answer for each questions**

**Aerobic exercises**

In the following we will ask you some questions **regarding the two exercises you just performed**. Please answer every question by crossing the box and do not skip any of these questions.

1. How good do you think this exercise is?

**Very Very**

**Bad** 0 0 0 0 0 0 0 **good**

2. How healthy do you think this exercise is?

**Very Very**

**unhealthy** 0 0 0 0 0 0 0 **healthy**

3. When you compare yourself to others of the same age and gender, do you think you are better, equal or worse in performing this exercise? Much worse – much better.

**Much Much**

**worse**  0 0 0 0 0 0 0 **better**

4. How did the exercise feel?

**Very Very**

**unpleasant** 0 0 0 0 0 0 0 **pleasant**

5. What did you think of the exercise?

**Very Very**

**boring** 0 0 0 0 0 0 0 **exciting**

6. After doing this exercise, I’m satisfied no matter what my performance is:

**Totally Totally**

**Disagree** 0 0 0 0 0 0 0 **agree**

**Please continue on the next page.**

7. The exercise I just did is something I would like to do in my free time:

**Totally Totally**

**Disagree** 0 0 0 0 0 0 0 **agree**

8. The exercise I just did is a good way to stay fit:

**Totally Totally**

**Disagree** 0 0 0 0 0 0 0  **agree**

9. I like the exercise because I get a benefit from it:

**Totally Totally**

**Disagree** 0 0 0 0 0 0 0  **agree**

10. Most people who are important to me would approve of me doing this exercise:

**Totally Totally**

**Disagree** 0 0 0 0 0 0 0  **agree**

11. This exercise is something most of my friends would do as well:

**Totally Totally**

**Disagree** 0 0 0 0 0 0 0  **agree**

12. I am not made for this exercise:

**Totally Totally**

**Disagree** 0 0 0 0 0 0 0 **agree**

13. This exercise did not feel right for me:

**Totally Totally**

**Disagree** 0 0 0 0 0 0 0 **agree**

**Thank you. Please continue on the next page.**

**Aerobic exercise in general**

The just performed task is an example for an aerobic exercise. Other aerobic exercises or activities are jogging/running, swimming, cycling and comparable activities. The following questions are about **aerobic exercises in general**.

14. To me aerobic exercises are… :

**Very Very**

**unimportant** 0 0 0 0 0 0 0 **important**

15. I am confident that, if I want to, I can do aerobic exercises for at least 3h per week (in total) on a regular basis:

**Not confident Very**

**at all**  0 0 0 0 0 0 0 **confident**

16. It is under my own control to perform aerobic exercises for at least 3h per week (in total) :

**Totally Totally**

**Disagree** 0 0 0 0 0 0 0  **agree**

17. How useful do you think aerobic exercises are?

**Not useful very**

**at all**  0 0 0 0 0 0 0 **useful**

18. How healthy are aerobic exercises for you?

**very very**

**unhealthy** 0 0 0 0 0 0 0 **healthy**

**Please continue on the next page.**

19. When you compare yourself to others of the same age and gender, do you think you are better, equal or worse performing aerobic exercises?

**Much Much**

**Worse** 0 0 0 0 0 0 0  **better**

20. I think aerobic exercises in general are:

**very very**

**unpleasant** 0 0 0 0 0 0 0 **pleasant**

21. I think aerobic exercises in general are:

**very very**

**boring** 0 0 0 0 0 0 0 **exciting**

22. I will do aerobic exercises in the future:

**very very**

**seldom** 0 0 0 0 0 0 0  **often**

23. I am planning to do aerobic exercises in the future:

**Not true Very**

**at all** 0 0 0 0 0 0 0 **true**

24. I expect to do aerobic exercises in the future:

**Totally Totally**

**Disagree** 0 0 0 0 0 0 0 **agree**

**Please continue on the next page.**

25. I will never be good at aerobic exercises:

**Totally Totally**

**Disagree** 0 0 0 0 0 0 0 **agree**

26. I would like to do aerobic exercises in my free time:

**Not true Very**

**at all** 0 0 0 0 0 0 0 **true**

27. I enjoy doing aerobic exercises:

**Not true Very**

**at all** 0 0 0 0 0 0 0 **true**

28. Aerobic exercises are a good way to stay fit:

**Totally Totally**

**Disagree** 0 0 0 0 0 0 0 **agree**

29. Aerobic exercises are a good way to show my performance to others:

**Not true Very**

**at all** 0 0 0 0 0 0 0 **true**

30. Most people who are important to me would approve of me doing aerobic exercises:

**Totally Totally**

**Disagree** 0 0 0 0 0 0 0  **agree**

31. Aerobic exercises are something most of my friends do as well:

**Totally Totally**

**Disagree** 0 0 0 0 0 0 0  **agree**

32. I am not suitable for aerobic exercises:

**Totally Totally**

**Disagree** 0 0 0 0 0 0 0  **agree**

**Please continue on the next page.**

**General questions**

33. How many times per week do you perform aerobic exercises? ______times.

34. How many hours (on average) do you perform aerobic exercises per week? ______ hours.

35. Would you like to improve your aerobic exercise performances?

**Absolutely**

**not** 0 0 0 0 0 0 0 **absolutely**

36. I especially like to perform in aerobic exercises because they are easy for me and I do not have to struggle a lot:

**Totally Totally**

**Disagree** 0 0 0 0 0 0 0 **agree**

**Thank you. Please wait for instructions**

**Resistance exercises**

In the following we will ask you some questions **regarding the two exercises you just performed**. Please answer every question by crossing the box and do not skip any of these questions.

1. How good do you think this exercise is?

**Very Very**

**Bad** 0 0 0 0 0 0 0 **good**

2. How healthy do you think this exercise is?

**Very Very**

**unhealthy** 0 0 0 0 0 0 0 **healthy**

3. When you compare yourself to others of the same age and gender, do you think you are better, equal or worse in performing this exercise? Much worse – much better.

**Much Much**

**worse** 0 0 0 0 0 0 0 **better**

4. How did the exercise feel?

**Very Very**

**unpleasant** 0 0 0 0 0 0 0 **pleasant**

5. What did you think of the exercise?

**Very Very**

**boring**  0 0 0 0 0 0 0 **exciting**

6. After doing this exercise, I’m satisfied no matter what my performance is:

**Totally Totally**

**Disagree** 0 0 0 0 0 0 0  **agree**

7. The exercise I just did is something I would like to do in my free time:

**Totally Totally**

**Disagree** 0 0 0 0 0 0 0  **agree**

**Please continue on the next page.**

8. The exercise I just did is a good way to stay fit:

**Totally Totally**

**Disagree** 0 0 0 0 0 0 0  **agree**

9. I like the exercise because I get a benefit from it:

**Totally Totally**

**Disagree** 0 0 0 0 0 0 0  **agree**

10. Most people who are important to me would approve of me doing this exercise:

**Totally Totally**

**Disagree** 0 0 0 0 0 0 0  **agree**

11. This exercise is something most of my friends would do as well:

**Totally Totally**

**Disagree** 0 0 0 0 0 0 0  **agree**

12. I am not made for this exercise:

**Totally Totally**

**Disagree** 0 0 0 0 0 0 0  **agree**

13. This exercise did not feel right for me:

**Totally Totally**

**Disagree** 0 0 0 0 0 0 0  **agree**

**Thank you. Please continue on the next page**

**Resistance exercise in general**

The just performed task is an example for resistance exercise. Other resistance exercises or activities are weight-lifting, working with your own body weight or comparable activities. The following questions are about **resistance exercises in general**.

14. To me resistance exercises are… :

**Very Very**

**unimportant** 0 0 0 0 0 0 0 **important**

15. I am confident that, if I want to, I can do resistance exercises for at least 3h per week (in total) on a regular basis:

**Not confident Very**

**at all**  0 0 0 0 0 0 0 **confident**

16. It is under my own control to perform resistance exercises for at least 3h per week (in total) :

**Totally Totally**

**Disagree** 0 0 0 0 0 0 0 **agree**

17. How useful do you think resistance exercises are?

**Not useful very**

**at all** 0 0 0 0 0 0 0 **useful**

18. How healthy are resistance exercises for you?

**very very**

**unhealthy** 0 0 0 0 0 0 0 **healthy**

**Please continue on the next page.**

19. When you compare yourself to others of the same age and gender, do you think you are better, equal or worse performing resistance exercises?

**Much Much**

**Worse**  0 0 0 0 0 0 0  **better**

20. I think resistance exercises in general are:

**very very**

**unpleasant** 0 0 0 0 0 0 0 **pleasant**

21. I think resistance exercises in general are:

**very very**

**boring**  0 0 0 0 0 0 0 **exciting**

22. I will do resistance exercises in the future:

**very very**

**seldom** 0 0 0 0 0 0 0  **often**

23. I am planning to do resistance exercises in the future:

**Not true Very**

**at all** 0 0 0 0 0 0 0 **true**

24. I expect to do resistance exercises in the future:

**Totally Totally**

**Disagree** 0 0 0 0 0 0 0  **agree**

**Please continue on the next page.**

25. I will never be good at resistance exercises:

**Totally Totally**

**Disagree** 0 0 0 0 0 0 0  **agree**

26. I would like to do resistance exercises in my free time:

**Not true Very**

**at all** 0 0 0 0 0 0 0 **true**

27. I enjoy doing resistance exercises:

**Not true Very**

**at all** 0 0 0 0 0 0 0 **true**

28. Resistance exercises are a good way to stay fit:

**Totally Totally**

**Disagree** 0 0 0 0 0 0 0  **agree**

29. Resistance exercises are a good way to show my performance to others:

**Not true Very**

**at all**  0 0 0 0 0 0 0 **true**

30. Most people who are important to me would approve of me doing resistance exercises:

Totally Totally

Disagree 0 0 0 0 0 0 0 agree

31. Resistance exercises are something most of my friends do as well:

**Totally Totally**

**Disagree** 0 0 0 0 0 0 0  **agree**

32. I am not suitable for resistance exercises:

**Totally Totally**

**Disagree** 0 0 0 0 0 0 0  **agree**

**Please continue on the next page.**

**General questions**

33. How many times per week do you perform resistance exercises? ______times.

34. How many hours (on average) do you perform resistance exercises per week? ______ hours.

35. Would you like to improve your resistance exercise performances?

**Absolutely**

**not** 0 0 0 0 0 0 0 **absolutely**

36. I especially like to perform in resistance exercises because they are easy for me and I do not have to struggle a lot:

**Totally Totally**

**Disagree** 0 0 0 0 0 0 0 **agree**

**Extra Question**

Which exercises did you like better?

**O** Chest press and leg press

**O** Cycling and running

What kind of exercise do you like better in general?

**O** Resistance exercise

**O** Aerobic exercise

What sport do you do in your free time?

**Thank you. Please wait for instructions**
