# Supplementary material for: A Benefit of Being Heavier Is Being Strong: a Cross-Sectional Study in Young Adults
Source: Sports Med Open. 2018 Mar 1;4:12. doi: 10.1186/s40798-018-0125-4 (PMC5833324; doi:10.1186/s40798-018-0125-4)
Supplement: Supplementary file 1 — READ ME – medical screening questionnaire for the study "A benefit of being heavier is being strong: a cross-sectional study in young adults". (ZIP 32 kb) [file 40798_2018_125_MOESM1_ESM.zip › Additional file 1. medscreening Q DUTCH.doc]

**Medische vragenlijst ter screening bij het onderzoek:**

***Een innovatieve methode om aerobe fitness te meten in een respiratiekamer***

**VERTROUWELIJK**

Contactgegevens:

Guy Plasqui

Humane Biologie, Universiteit Maastricht

PO Box 616, 6200 MD Maastricht

Tel 043-3881506

Email: g.plasqui@maastrichtuniversity.nl

Deze vragenlijst is bedoeld om na te gaan of er persoonlijke kenmerken of gezondheidsaspecten zijn die deelname aan het onderzoek uitsluiten, of die de resultaten van het onderzoek zouden kunnen beïnvloeden.

Alle gegevens in deze vragenlijst zullen strikt vertrouwelijk behandeld worden.

De vragenlijst bestaat uit:

A. Persoonlijke kenmerken
B. Leefgewoonten
C. Persoonlijke gezondheid
D. Familiaire gezondheid

**A. Persoonlijke kenmerken**

Proefpersoon nummer

Lengte:

Gewicht:

Leeftijd:

Geslacht:	m / v

**B. Leefgewoonten**

Roken

1. Rookt u

O JA

Zo ja, hoeveel sigaretten per dag:

O NEE

Medicijnen

2. Gebruikt u medicijnen

O NEE

O JA

Zo ja, welke en dosering (gelieve alle medicatie, ook pijnstillers ed. in frequentie te vermelden):

Lichamelijke activiteit *(omcirkel uw keuze)*

*Werk*

3. Wat is uw (hoofd)beroep
4. Tijdens mijn werk zit ik...	nooit / zelden / soms / vaak / altijd
5. Tijdens mijn werk sta ik...	nooit / zelden / soms / vaak / altijd
6. Tijdens mijn werk loop ik...	nooit / zelden / soms / vaak / altijd
7. Tijdens mijn werk til ik

zware voorwerpen...	nooit / zelden / soms / vaak / altijd

8. Tijdens mijn werk zweet ik...	nooit / zelden / soms / vaak / altijd
9. Na mijn werk ben ik moe...	nooit / zelden / soms / vaak / altijd
10. In vergelijking met andere mensen van mijn leeftijd is volgens mij mijn werk lichamelijk...

veel zwaarder / zwaarder / net zo zwaar / lichter / veel lichter

*Vrije tijd*

11. In mijn vrije tijd zweet ik...	nooit / zelden / soms / vaak / altijd
12. In mijn vrije tijd sport ik...	nooit / zelden / soms / vaak / altijd
13. In mijn vrije tijd loop ik...	nooit / zelden / soms / vaak / altijd
14. In mijn vrije tijd fiets ik...	nooit / zelden / soms / vaak / altijd
15. In mijn vrije tijd kijk ik televisie..	nooit / zelden / soms / vaak / altijd
16. In vergelijking met andere mensen van mijn leeftijd is mijn lichamelijke activiteit in mijn vrije tijd volgens mij...

veel hoger / hoger / hetzelfde / lager / veel lager

17. Hoeveel minuten loopt of fietst u per dag naar en van uw werk en om boodschappen te doen...

minder dan 5 / 5-15 / 15-30 / 30-45 / meer dan 45

18. Doet u aan sport

O JA

O NEE (ga door naar vraag 21)

19. Welke sport beoefent u het vaakst

Hoeveel uur per week	minder dan 1 / 1 / 1.5 / 2 / 2-3 /

3-4 / meer dan 4

Hoeveel maanden per jaar	minder dan 1 / 1-3 / 4-6 / 7-9 /

meer dan 9

20. Als u een tweede sport beoefent

Welke sport is dit

Hoeveel uur per week	minder dan 1 / 1 / 1.5 / 2 / 2-3 /

3-4 / meer dan 4

Hoeveel maanden per jaar	minder dan 1 / 1-3 / 4-6 / 7-9 /

meer dan 9

**C. Persoonlijke gezondheid**

21. Bent u in goede gezondheid

O JA

O NEE

22. Voelt u zich gewoonlijk gezond

O JA

O NEE

23. Bent u nu onder medische behandeling

O NEE

O JA

Zo ja, bij huisarts of specialist? ....................................................................

Waarvoor? …………………………………………………………………

24. Geeft u a.u.b. aan of u lijdt, of ooit heeft geleden aan een van onderstaande ziekten, klachten of symptomen. Zo ja, wilt u dan het jaartal erachter vermelden

O hartinfarct

O overige hart- of vaatziekten

O beroerte of hersenbloeding

O hoge bloeddruk

O longziekte

O verhoogd cholesterolgehalte in het bloed

O allergische reactie op voedsel

O allergische reactie op andere stoffen

O lever aandoeningen

O astma of (chronische) bronchitis

O suiker of eiwit in de urine

O suikerziekte

O schildklierziekte

O bloedarmoede

O eetstoornissen

O nierziekte

O maag- of darmziekten

O psychiatrische ziekten

O andere, namelijk:

25. Heeft u wel eens pijn of een onaangenaam gevoel op of in de borst gehad (klachten bij verkoudheid niet meegerekend)

O JA

Zo ja, waar:	midden / links / links met arm en of

kaak / rechts

Hoe lang duurde het:	< 1 min / 1-10 min / > 10 min

Wat was u aan het doen: …………………………….

O NEE

26. Kunt u zonder problemen/pijn fietsen?

O JA

O NEE

27. Heeft u wel eens last van kortademigheid in rust

O JA

O NEE

28. Heeft u wel eens last van kortademigheid tijdens inspanning

O JA

Zo ja, hoeveel trappen kunt u nog oplopen voordat u stil

moet staan:......................

O NEE

29. Heeft u wel eens last van nachtelijke kortademigheid

O JA

O NEE

**D. Familiaire gezondheid**

30. Geeft u a.u.b. aan of bij uw familie (vader, moeder, broers, zussen, kinderen) een van de volgende ziekten voorkomt:

O hartinfarct

zo ja, op welke leeftijd…………………….

O hoge bloeddruk

O verhoogd cholesterolgehalte in het bloed

O suikerziekte (diabetes mellitus)

O shildklierziekten

O astma of (chronische) bronchitis

O maag- of darmziekten

O zwaarlijvigheid

**- Hartelijk dank voor uw medewerking -**
